# The differences of poor SRH among municipalities in Iwate after the Great East Japan Earthquake

**DOI:** 10.1038/s41598-021-96237-3

**Published:** 2021-08-26

**Authors:** Shuko Takahashi, Haruki Shimoda, Kiyomi Sakata, Akira Ogawa, Seiichiro Kobayashi, Ichiro Kawachi

**Affiliations:** 1 Division of Medical Education, Iwate Medical University, 1-1-1, Idaidori, Yahaba-cho, Shiwa-gun, Iwate, 028-3694 Japan; 2 Takemi Program in International Health, Harvard T.H. Chan School of Public Health, 665 Huntington Avenue, Building 1, Room 1210A, Boston, MA 02115 USA; 3 Department of Hygiene and Preventive Medicine, School of Medicine, Iwate Medical University, 1-1-1, Idaidori, Yahaba-cho, Shiwa-gun, Iwate, 028-3694 Japan; 4 Iwate Medical University, 1-1-1, Idaidori, Yahaba-cho, Shiwa-gun, Iwate, 028-3694 Japan; 5 Department of Social and Behavioral Sciences, Harvard. T.H. Chan School of Public Health, 401 Park Drive, Boston, MA 02215 USA

**Keywords:** Epidemiology, Population screening

## Abstract

The health of communities has been observed to recover at differential rates in the wake of disasters. In the present study, the 5-year trends in poor self-rated health (SRH) in three municipalities of Iwate Prefecture following the 2011 Great East Japan Earthquake and Tsunami were compared. Annual surveys were conducted from 2011 to 2015 in three municipalities (Otsuchi, Rikuzentakata, and Yamada) that were heavily damaged by the tsunami. We tracked the prevalence of poor SRH in 10,052 participants (mean age, 61.0 years; 39.0% men). Trends in the prevalence of poor SRH were analyzed using generalized linear mixed effect models with control for covariates. Immediately after the disaster (2011), all three municipalities reported similar prevalences of poor SRH (around 15%). Among people under the age of 65 years, there was a gradual improvement in health for residents of Rikuzentakata and Yamada, but the prevalence of poor SRH remained persistently high in Otsuchi. Among people over the age of 65 years, the prevalence of poor SRH remained constant in Rikuzentakata and Yamada but increased over a 5-year follow-up period in Otsuchi. The delayed health recovery in Otsuchi may be due to the characteristics of the local health system. Examination of the variations in health recovery may provide clues about the sources of disaster resilience.

## Introduction

Ten years have passed since the 2011 Great East Japan Earthquake that devastated coastal cities in the Tohoku region of Japan. Iwate Prefecture, which is located close to the earthquake epicenter, was one of the most severely affected areas, reporting a death toll of 4695 and 1122 missing. Damage was greater in the southern coastal cities and towns in Iwate Prefecture, and many health care facilities and city offices in those areas were destroyed by the tsunami. There has been an exodus of medical and welfare providers, resulting in no medical care in some areas^1^.

Survivors of the disaster experienced difficult conditions for several years due to loss of property and deaths of relatives^2–5^. An important topic in disaster research is to understand the variability in recovery following a major disaster. The ability to withstand shocks and to recover quickly is broadly defined as disaster resilience. Disaster resilience at the individual level is determined by various factors including pre-disaster health status, severity of trauma experienced during the disaster, and availability of social support and other resources in the aftermath of the disaster. At the community level, disaster resilience is affected by various factors including the level of preparedness in the community, strength of the health system to deal with mass emergencies, and level of resources available during the long recovery phase. Descriptive studies of this type are the first step toward gaining a better understanding of disaster resilience. To the best of our knowledge, however, few studies were carried out in the aftermath of the 2011 Japan Earthquake & Tsunami to determine community-level variations in rates of health recovery. Previous studies have shown poor health status (including poor self-rated health: SRH) among survivors following natural disasters^6,7^. Differences in SRH depending on differences in severity of housing damage and living conditions after the disaster have been reported^8,9^. However, those studies focused on variations in individual health status, and there has been no study in which differences in health status among municipalities were evaluated.

Accordingly, the aim of this study was to document the trajectories of health status among residents of three municipalities in Iwate Prefecture over a period of 5 years following the 2011 Great East Japan Earthquake.

## Results

We compared characteristics of the analytic sample at baseline (2011) and the whole population in 2011 in three municipalities (Supplementary Table 1). In the present study, the percentage of women was higher than men. While the percentages of people in several age groups (including people aged from 20 to 59 years) were higher in the 2011 local census, the percentage of people aged 60 years or more was higher in our analytic sample.

Table 1 shows the baseline characteristics of participants in the 2011 survey. While the prevalence of poor SRH was high in Otsuchi among participants aged 64 years or younger, the prevalences of poor SRH were not significantly different in the three municipalities among participants aged 65 years or older. The mean ages of participants in Rikuzentakata and Otsuchi were older than the mean age of participants in Yamada (mean ages: 61.5 years in Otsuchi, 62.4 years in Rikuzentakata, and 58.4 years in Yamada). Despite similar levels of SRH at baseline, there were differences in the characteristics of residents among the localities. For example, in Otsuchi, there were significantly higher percentages of people living in prefabricated temporary housing, people who were unemployed and people who were obese. In Yamada, there were significantly higher percentages of men, drinkers, people reporting insomnia, people with a lower level of social capital, and people without comorbid illnesses. Among participants aged 65 years or older, people in Rikuzentakata were significantly older and people in Yamada were significantly younger than those in Otsuchi (mean ages: 73.0 years in Otsuchi, 73.7 years in Rikuzentakata, and 71.8 years in Yamada).

**Table 1:** Baseline characteristics of participants in the 2011 and survey (n = 10,052).

	Missing	64 years or younger (n = 5327)					Missing	65 years or older (n = 4725)				
		Otsuchi (n = 1054)	Rikuzentakata (n = 2351)	Yamada (n = 1922)	*P* value	Post hoc test		Otsuchi (n = 995)	Rikuzentakata (n = 2513)	Yamada (n = 1217)	*P* value	Post hoc test
	n (%)	Mean (SD)/n (%)	Mean (SD)/n (%)	Mean (SD)/n (%)			n (%)	Mean (SD)/n (%)	Mean (SD)/n (%)	Mean (SD)/n (%)		
Age, years	0 (0.0)	50.7 (11.6)	50.5 (11.4)	50.0 (11.7)	0.211		0 (0.0)	73.0 (5.7)	73.7 (5.6)	71.8 (4.8)	< 0.001*	^a,b,c^
**Sex**												
Men	0 (0.0)	342 (32.4)	814 (34.6)	736 (38.3)	0.003*		0 (0.0)	434 (43.6)	1059 (42.1)	540 (44.4)	0.398	
**Functional disability**												
General frailty							332 (7.0)	63 (6.9)	115 (4.9)	52 (4.6)	0.034*	
Lower IADL							57 (1.2)	268 (27.3)	454 (18.3)	292 (24.2)	< 0.001*	
Low physical strength							113 (2.4)	171 (17.7)	485 (19.8)	196 (16.4)	0.042*	
Malnutrition							46 (1.0)	299 (30.5)	599 (24.0)	376 (31.3)	< 0.001*	
Low oral function							60 (1.3)	137 (14.0)	405 (16.3)	172 (14.3)	0.143	
Home-bound							8 (0.2)	110 (11.1)	275 (11.0)	141 (11.6)	0.828	
Cognitive impairment							58 (1.2)	424 (43.1)	1078 (43.4)	509 (42.4)	0.838	
**Living conditions**												
Living in temporary housing	88 (1.7)	396 (38.3)	799 (34.3)	507 (27.0)	< 0.001*		81 (1.7)	350 (35.9)	752 (30.4)	354 (29.5)	0.003*	
**Socioeconomic status**												
Severely distressed economic situation	8 (0.2)	600 (57.0)	1410 (60.0)	1128 (58.9)	0.27		26 (0.6)	430 (43.5)	1092 (43.6)	506 (42.0)	0.655	
Unemployment (2011)	189 (3.5)	281 (28.3)	580 (25.4)	437 (23.5)	0.019*		126 (2.7)	80 (8.6)	263 (10.6)	210 (17.8)	< 0.001*	
**Health habits**												
Current smokers	1 (0.0)	262 (24.9)	523 (22.2)	453 (23.6)	0.226		0 (0.0)	100 (10.1)	214 (8.5)	97 (8.0)	0.201	
Drinkers	1 (0.0)	394 (37.4)	816 (34.7)	757 (39.4)	0.006*		0 (0.0)	296 (29.7)	667 (26.5)	375 (30.8)	0.013*	
**Psychological factors**												
Moderate psychological distress	66 (1.2)	415 (40.1)	898 (38.6)	774 (40.7)	0.226		98 (2.1)	326 (33.6)	799 (32.4)	392 (32.9)	0.197	
Severe psychological distress		81 (7.8)	148 (6.4)	137 (7.2)				52 (5.4)	123 (5.0)	81 (6.8)		
PTSD Symptom	36 (0.7)	445 (42.7)	938 (40.2)	696 (36.3)	0.002*		44 (0.9)	462 (47.0)	1239 (49.8)	531 (43.9)	0.003*	
Insomnia	58 (1.1)	386 (36.9)	750 (32.2)	711 (37.5)	0.001*		79 (1.7)	289 (29.8)	739 (29.8)	410 (34.3)	0.016*	
**Social factors**												
Low level of social network	87 (1.6)	461 (44.7)	1023 (44.2)	877 (46.3)	0.409		121 (2.6)	383 (39.7)	886 (36.1)	460 (38.7)	0.097	
Low level of social capital	12 (0.2)	134 (12.7)	190 (8.1)	246 (12.8)	< 0.001*		20 (0.4)	93 (9.4)	191 (7.6)	123 (10.1)	0.024*	
**Disease factors**												
Having present illness	0 (0.0)	536 (50.9)	1223 (52.0)	888 (46.2)	0.001*		0 (0.0)	804 (80.8)	2043 (81.3)	915 (75.2)	< 0.001*	
Having symptoms	60 (1.1)	512 (49.3)	1155 (49.6)	879 (46.2)	0.067		85 (1.8)	365 (37.7)	1120 (45.3)	505 (42.2)	< 0.001*	
**Cardiovascular risk factors**												
Obesity	13 (0.2)	367 (34.9)	709 (30.2)	594 (31.0)	0.024*		332 (7.0)	397 (39.9)	796 (31.7)	416 (34.2)	< 0.001*	
Diabetes mellitus	0 (0.0)	69 (6.5)	163 (6.9)	131 (6.8)	0.918		113 (2.4)	134 (13.5)	341 (13.6)	153 (12.6)	0.690	
Metabolic syndrome	14 (0.3)	221 (21.0)	530 (22.6)	400 (20.9)	0.337		46 (1.0)	312 (31.4)	785 (31.2)	360 (29.6)	0.545	
**Self-rated health**												
Poor self-rated health	0 (0.0)	166 (15.7)	299 (12.7)	286 (14.9)	0.030		332 (7.0)	165 (16.6)	418 (16.6)	202 (16.6)	0.999	

Continuous variables are presented as means (standard deviations). Categorical variables are presented as the number of cases (%).

*P* values were calculated using analysis of variance for continuous variables using Bonferroni correction, and the chi-squared test for categorical variables.

*SD* standard deviation, *IADL* instrumental activities of daily living, *PTSD* post-traumatic stress disorder.

*Statistically significant differences among municipalities.

^a^Statistically significant difference between Otsuchi and Rikuzentakata.

^b^Statistically significant difference between Rikuzentakata and Yamada.

^c^Statistically significant difference between Yamada and Otsuchi.

**Figure 1:**
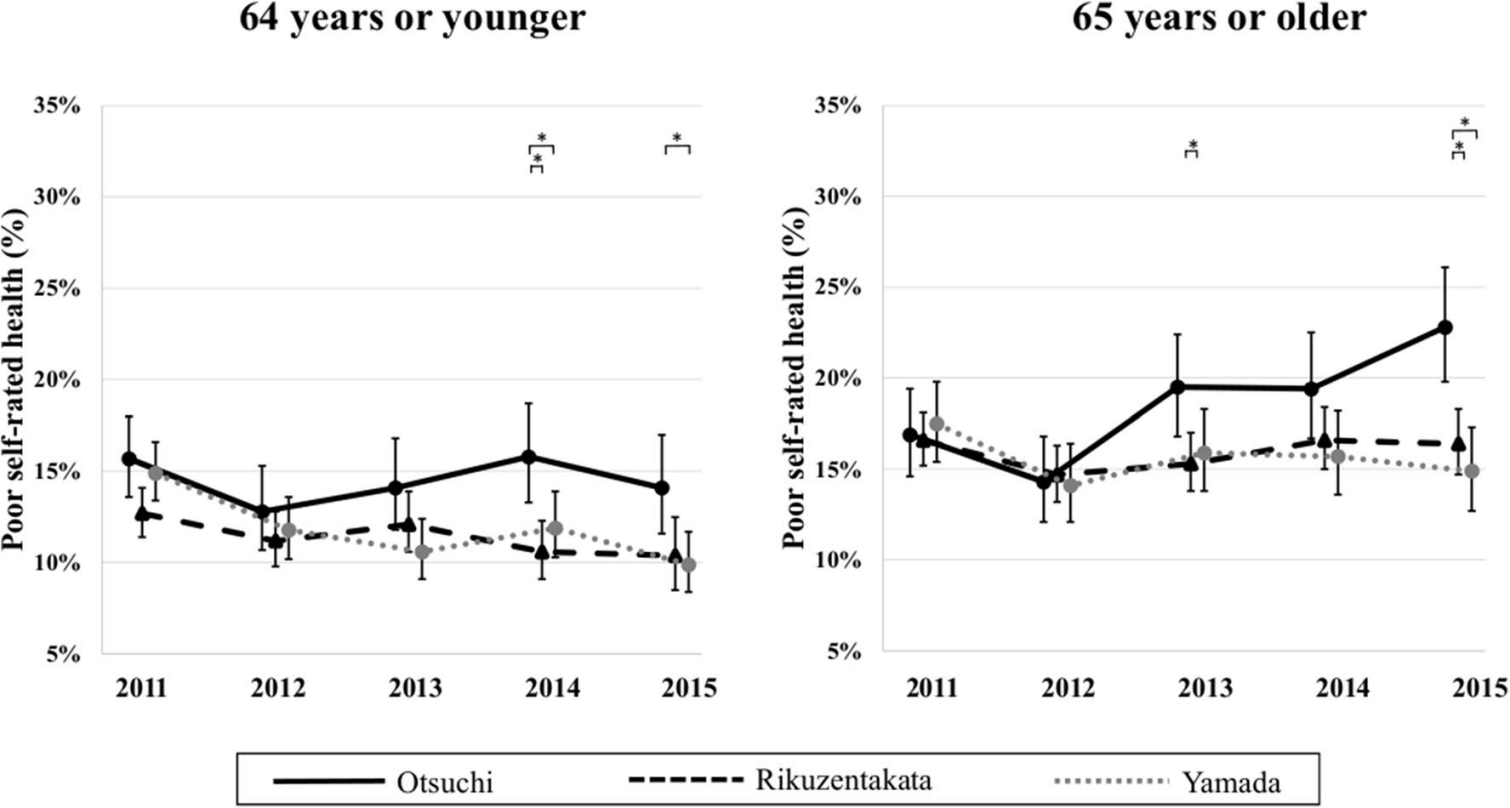
Trends in estimated marginal means of the prevalence ratios of poor self-rated health among municipalities from 2011 to 2015. *Statistically significant (*p*-value < 0.05). Error bars represent 95% confidence intervals.

Table 2 shows the odds ratios (ORs) for poor SRH during the survey period among municipalities estimated from the generalized mixed effect models. The number of participants indicates individuals who participated in at least one survey from 2011 to 2015 and had all variables adjusted in each model in the survey in which they participated. Figure 1 shows the trend in the estimated marginal mean of prevalence of poor SRH from 2011 to 2015 adjusted for age and sex. Otsuchi residents appeared to be less healthy than residents of the other communities, even though the prevalences of poor SRH were similar at baseline. In people aged 64 years or younger, although there was no significant difference in the prevalences of poor SRH in 2011 among the three municipalities, the prevalences of poor SRH decreased in Rikuzentakata and Yamada, while Otsuchi had a persistently elevated prevalence of poor SRH. Among people aged 65 years or older, although the prevalences of poor SRH were not significantly different among the three municipalities at baseline (in 2011), the prevalence of poor SRH significantly increased in Otsuchi during the follow-up period, while the prevalences of poor SRH remained constant in Rikuzentakata and Yamada. Hence, Otsuchi was different from the other towns with regard to its slow pace of recovery. In multi-variable regression models, we controlled for additional health indicators to see whether they could partially explain the disparities in SRH that emerged during the follow-up period between Otsuchi and the two other communities. In Model 1 plus functional disability in people aged 65 years or older (Model 2), the ORs for poor SRH in Otsuchi and Rikuzentakata increased and were significantly higher than those in Yamada from 2014 to 2015 (Otsuchi in 2015: OR [95% confidence interval (CI)] 1.90 [1.38 to 2.64], *P* < 0.001; Rikuzentakata in 2015: odds OR [95% CI] 1.37 [1.05 to 1.81], *P* = 0.024).We next adjusted for all characteristics that predict poor SRH in an attempt to explain the differences in SRH that we observed (Model 3). We show the ORs of poor SRH adjustment for variables such as in present illness, symptoms, and general frailty scores in the Kihon Checklist which were converted from categorical variables into discrete variables (Supplementary Table 2). For people aged 65 years or older, the OR for poor SRH in Otsuchi remained significantly higher than that in the other localities, even after adjusting for the full range of potential confounders (Otsuchi in 2015: OR [95% confidence interval (CI)] 1.70 [1.11 to 2.60]; *P* = 0.002).

**Table 2:** Comparison of poor self-rated health among municipalities using the generalized mixed effect models.

	64 years or younger				65 years or older					
	Model 1 (n = 5327)		Model 3 (n = 5052)		Model 1 (n = 4725)		Model 2 (n = 4662)		Model 3 (n = 4481)	
	Odds ratio	95% CI	Odds ratio	95% CI	Odds ratio	95% CI	Odds ratio	95% CI	Odds ratio	95% CI
Intercept	0.14	0.11, 0.19	0.01	0.01, 0.02	0.02	0.01, 0.05	0.11	0.05, 0.24	0.02	0.01, 0.04
Age	1.01	1.00, 1.01	1.00	0.99, 1.01	1.03	1.02, 1.04	1.00	0.99, 1.01	1.00	0.98, 1.01
Sex (men)	0.90	0.79, 1.02	1.18	1.00, 1.39	0.87	0.77, 0.98	1.01	0.89, 1.14	1.57	1.34, 1.84
Otsuhi (ref; Yamada)	1.06	0.86, 1.31	1.03	0.80, 1.32	0.96	0.76, 1.20	0.94	0.74, 1.20	0.99	0.74, 1.34
Rikuzentakata	0.83	0.70, 0.99	0.83	0.68, 1.02	0.94	0.78, 1.13	0.98	0.80, 1.19	0.98	0.78, 1.24
2011	Base		Base		Base		Base		Base	
2012	0.76	0.64, 0.91	0.91	0.71, 1.16	0.78	0.65, 0.93	0.76	0.61, 0.94	0.80	0.60, 1.08
2013	0.68	0.57, 0.82	0.85	0.66, 1.09	0.89	0.74, 1.08	0.98	0.78, 1.22	1.04	0.78, 1.40
2014	0.77	0.64, 0.94	1.14	0.89, 1.46	0.88	0.73, 1.07	0.91	0.73, 1.13	1.03	0.78, 1.37
2015	0.63	0.51, 0.77	0.93	0.72, 1.20	0.82	0.67, 1.01	0.88	0.70, 1.12	1.04	0.77, 1.40
2012 × Otsuchi (ref; 2011 × Yamada)	1.04	0.79, 1.37	0.91	0.61, 1.35	1.06	0.81, 1.39	1.21	0.88, 1.66	1.21	0.78, 1.88
2012 × Rikuzentakata	1.14	0.90, 1.44	1.17	0.85, 1.61	1.11	0.89, 1.39	1.32	1.02, 1.72	1.32	0.94, 1.86
2013 × Otsuchi	1.30	0.98, 1.74	1.40	0.95, 2.08	1.33	1.00, 1.77	1.50	1.10, 2.07	1.40	0.92, 2.14
2013 × Rikuzentakata	1.40	1.09, 1.80	1.42	1.03, 1.97	1.02	0.81, 1.29	1.20	0.92, 1.56	1.13	0.81, 1.58
2014 × Otsuchi	1.31	0.98, 1.75	1.35	0.93, 1.96	1.35	1.01, 1.79	1.54	1.11, 2.12	1.44	0.95, 2.19
2014 × Rikuzentakata	1.12	0.87, 1.46	1.08	0.78, 1.50	1.14	0.90, 1.43	1.50	1.15, 1.95	1.39	1.00, 1.92
2015 × Otsuchi	1.41	1.03, 1.92	1.10	0.73, 1.64	1.77	1.32, 2.37	1.90	1.38, 2.64	1.70	1.11, 2.60
2015 × Rikuzentakata	1.30	0.99, 1.71	1.20	0.86, 1.67	1.20	0.94, 1.53	1.37	1.05, 1.81	1.22	0.87, 1.71
General frailty							1.34	1.08, 1.68	1.28	0.98, 1.68
Low levels of IADL							1.20	1.07, 1.35	1.21	1.05, 1.40
Low level of physical strength							2.28	2.02, 2.57	2.00	1.75, 2.29
Malnutrition							1.46	1.31, 1.62	1.30	1.15, 1.48
Low level of oral function							1.56	1.39, 1.75	1.21	1.06, 1.38
Home-bound							1.08	0.91, 1.28	1.03	0.84, 1.26
Cognitive impairment							1.28	1.16, 1.40	1.08	0.96, 1.21
Living in prefabricated temporary housing			1.01	0.88, 1.15					0.99	0.87, 1.14
Severely distressed economic situation			1.19	1.05, 1.35					1.14	1.01, 1.28
Unemployment (2011)			0.80	0.69, 0.92					0.72	0.58, 0.88
Current smokers			1.28	1.09, 1.50					1.02	0.81, 1.29
Drinkers			0.83	0.72, 0.96					0.80	0.67, 0.94
Moderate psychological distress			1.68	1.49, 1.91					1.61	1.43, 1.82
Severe psychological distress			3.27	2.63, 4.06					2.19	1.74, 2.75
PTSD symptom			1.04	0.92, 1.18					1.06	0.95, 1.19
Insomnia			2.43	2.14, 2.75					2.14	1.90, 2.42
Low level of social network			1.12	1.00, 1.26					1.20	1.08, 1.34
Low level of social capital			1.18	1.01, 1.38					1.11	0.95, 1.30
Having present illness			2.15	1.89, 2.44					1.90	1.57, 2.29
Having symptoms			5.20	4.55, 5.94					4.85	4.29, 5.48
Obesity			0.95	0.82, 1.10					0.95	0.84, 1.09
Diabetes mellitus			1.26	1.02, 1.55					1.14	0.96, 1.34
Metabolic syndrome			1.19	1.02, 1.37					1.00	0.87, 1.14

*CI* confidence interval, *IADL* instrumental activities of daily living, *PTSD* post-traumatic stress disorder.

The number of participants represents the individuals who participated in at least one survey from 2011 to 2015 and had all variables adjusted in each model in one's participated survey. Because people aged 64 years or younger did not have data about functional disability, there was not model 2 in the people aged 64 years or younger.

To consider possible selection bias caused by missing values, we calculated the ORs for people with missing values and those without missing values in the 2011 survey using inverse propensity weighting in the generalized mixed effect models (Supplementary Table 3). People aged 65 years or older in Otsuchi tended to have higher ORs from 2013 to 2015 than those for people aged 65 years or older in Yamada, but the differences were not significant.

## Discussion

In this study, we tracked the post-disaster heath status of survivors in three localities that were severely damaged by the 2011 earthquake and tsunami. Despite starting at baseline with similar levels of health, the survivors had significantly different trajectories of health in the aftermath of the disaster. Survivois aged under 65 years in Otsuchi reported persistently high rates of poor SRH and there was a rising trend in poor health in survivors aged over 65 years. The variables that we adjusted for in Model 2 and Model 3 partially explained the disparity in poor SRH between municipalities, viz., functional disability, living conditions, self-assessed economic situation, employment status, health habits, psychosocial factors, and disease markers in people aged 65 years or older. Importantly, differences in the prevalence of poor health between the three localities could not be accounted for by differences in the predictors of poor health including functional disability, economic situation, employment status after the disaster, health habits, psychosocial factors, social connectedness, and comorbid diseases.

Factors predicting poor SRH after a natural disaster were investigated in previous studies. Demirchyan et al. showed in a prospective study (n = 725) that baseline BMI, multi-morbidity, perceived poor living standards, and number of stressful life events were each associated with poor SRH for a period of up to 23 years after the Armenia earthquake^6^. Ruggiero et al. examined the relationships of SRH with disaster characteristics, social resources, and post-disaster outcomes among 1425 adults residing in 33 counties in Florida after the 2004 Florida hurricanes^7^. They found that poor SRH was associated with older age, extreme fear during the hurricane, low level of social support, and depression. However, area differences in poor SRH were not the focus of previous studies.

There have been two studies in which the trajectory of poor SRH after a natural disaster was traced. Tsubota et al. investigated the association between health risks and frailty in the elderly for 5 years after the Great East Japan Earthquake (n = 2261)^9^. People who had extensive damage to their houses had significantly poor SRH compared to that in people who had partial or no damage to their homes. Kusama et al. investigated the trajectories of SRH for 7 years following the Great East Japan Earthquake in a repeated cross-sectional study (n = 179,255)^8^. Although the annual number of people who reported poor SRH did not change among residents living in prefabricated temporary housing, the highest prevalence of poor SRH was found among residents living in public housing. Those two studies were similar to our study in terms of focusing on the long-term trajectory of SRH in disaster-affected areas. However, the previous studies focused on individual trajectories of SRH, while our study focused on differences in the prevalence of poor SRH from an ecological (community) perspective.

**Figure 2:**
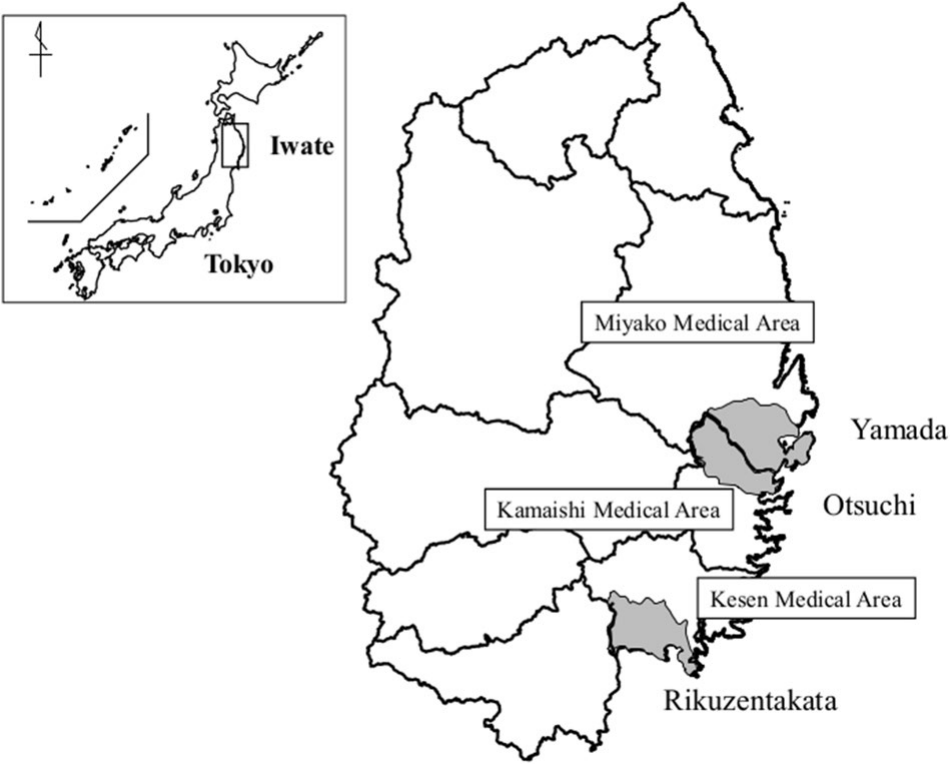
Map of the study area and secondary medical areas in Iwate Prefecture (created by MANDARA version 9.4.1. http://ktgis.net/lab/index.php). The black square shows the study area along the Pacific Ocean coast. The municipalities included in the study (Otsuchi, Rikuzentakata, and Yamada) are marked in dark gray. Secondary medical areas in Iwate Prefecture are shown by solid lines. Otsuchi, Rikuzentakata, and Yamada are included in Kamaishi, Kesen and Miyako medical areas, respectively.

Although the precise reasons for the variations in prevalence of poor SRH among the municipalities could not be explained by the extensive range of variables we collected in our study, there are some possible explanations based on factors that we did not capture. First, there was variation in the distribution of mortality between the municipalities (Table 3). The fatality rate in men was higher in Otsuchi than in the other two municipalities (51.0% in Otsuchi, 45.1% in Rikuzentakata and 45.0% in Yamada). Since women tend to report worse SRH than men^10^, the high fatality rate in men might have contributed to the high prevalence of poor SRH in Otsuchi. However, if gender differences were responsible for the differences in SRH between localities, the disparity in SRH should have disappeared after controlling for sex. This was not the case. Second, living conditions may have been associated with dementia and IADL. When we compared the characteristics of participants in the 2015 follow-up survey among the municipalities, the percentages of people living in temporary housing, people with insomnia, people with a low level of social network, people with a low level of IADL, people who were home-bound and people with dementia were all significantly higher in Otsuchi than in the other two towns (P < 0.001 in all factors except for low level of social network, P = 0.002) (Supplementary Table 4). Following the disaster, victims who lost their homes were evacuated to shelters and then moved to prefabricated temporary housing (resembling FEMA trailer homes). Subsequently, some people were relocated to permanent public housing, while others built their own homes at higher elevations^11^. Because Otsuchi did not have enough land space to accommodate new housing for displaced survivors, more of them were forced to live for longer in the temporary housing. Because the quality of housing was poor in these trailer-style homes, people in Otsuchi were forced to put up with worse living conditions for a longer time than were people in neighboring areas. The higher percentages of people with insomnia, social isolation, and cognitive decline in Otsuchi may be indicative of the strains associated with living in temporary housing. Third, the disaster might have caused damage to human resources in Otsuchi. With regard to long-term care of the elderly, each municipality was instructed to establish a system of cooperation between health care workers, medical institutions and nursing care institutions as well as to secure human resources including social workers and long-term care specialists. Local government officials and public health officers (employed by the municipalities) therefore played a critical role in securing these services for the elderly. In the 2011 disaster, Otsuchi had the highest percentage of casualties (dead or missing) among administrative officials due to the destruction of their town hall (Table 3). Indeed by 2015, there were fewer care managers (i.e., individuals who coordinate long-term care services for the elderly) in Otsuchi than in Yamada. The loss of these officials could have contributed to the less efficient delivery of services to the elderly (numbers (percentages) of dead or missing who worked in damaged city offices: 40 (28%) in Otsuchi, 111 (25%) in Rikuzentakata and 2 (1%) in Yamada). (Table 3). The problems in Otsuchi were compounded by the fact that the mayor died on the day of the disaster, resulting in a temporary leadership crisis. In contrast, staff at the Iwate Prefectural Takata Hospital (the main hospital in the neighboring city of Rikuzentakata) managed to re-establish home visiting/care services at an early stage after the disaster because they had been focusing on such services even before the disaster^1^. Through a combination of preparedness and strong leadership, Rikuzentakata succeeded in minimizing the disruption to services despite the small number of care managers. Fourth, the extent of damage to health care facilities varied by locality. Iwate Prefecture has 14 cities, 15 towns, and 4 villages in 9 secondary medical areas. Otsuchi, Rikuzentakata, and Yamada are served by the Kamaishi, Kesen, and Miyako medical areas, respectively (Fig. 2). Kamaishi (Otsuchi) area had the problem of a small number of medical doctors before the disaster. Kamaishi area also had a smaller number of clinics after the disaster than the numbers of clinics in Kesen (Rikuzentakata) and Miyako (Yamada) areas (Table 3). Furthermore, Iwate Prefectural Kamaishi Hospital (Kamaishi secondary medical area), which is the only hospital providing tertiary care, was partially destroyed by the earthquake. Many inpatients were transferred to other hospitals in inland areas, and admission was restricted for several months after the disaster^1^. Residents of Otsuchi may have therefore received fewer medical services than people living in the other two municipalities. Fifth, surge capacity and coordination of services within secondary medical areas should be considered. To deal with population aging and decline, the Japanese government has been engaged in a long-term initiative to centralize the delivery of healthcare services in less populous regions—known as the “*chiiki houkatsu care* system”^12^. Under this initiative, local municipalities are responsible for designating “secondary medical areas” where older patients can be transferred to nearby tertiary care institutions in case of acute exacerbations in demand, such as disasters. The system encourages cooperation among providers involved in home medical care and nursing care facilities after they are discharged from medical institutions. In our study, the disparity in poor SRH widened in Otsuchi compared to that in Rikuzentakata after adjusting for functional disability. We speculate that the community-based comprehensive care system may not have functioned effectively in the Kamaishi secondary medical area (which includes Otsuchi). For example, patients may not have been directed to medical care facilities appropriately, e.g., presenting to clinics that lacked sufficient capacity to care for severely ill patients after the disaster^13^. In addition, after being discharged from hospitals, someone suffering from functional disability may not have received appropriate services such as home rehabilitation. Public data show that the number of long-term care cases per 100,000 persons was highest in the Kamaishi secondary medical area among the three medical areas in 2015; the number of cases with mild functional disability was the highest in that area (Table 3). However, the number of older people with a mild functional disability who were receiving services was smaller in Otsuchi Town than in Kamaishi City (both located within the Kamaishi secondary medical area), and there was a notable difference between the numbers of people receiving services in those two municipalities (number of people receiving services per 100,000 people with mild functional disability: Otsuchi, 4623.6 vs. Kamaishi, 6385.9). Hence, resources for long-term care services may not have been equitably distributed within the Kamaishi secondary medical area.

**Table 3:** Difference of data across the disaster among three municipalities.

		Otsuchi	Rikuzentakata	Yamada
Size	Size (km^2^)	200.42	231.94	262.81
Population	Before the disaster (2010)	15,276	23,300	18,617
	Immediately after the disaster (2011)	12,681	20,252	16,903
	5 years after the disaster (2015)	11,642	19,473	15,578
	Population density (2015)	58.1	84	59.3
Households	The number of households (2010)	5689	7785	6605
	The number of households (2015)	5447	7601	6712
Sex	The number of men (2015)	5723	9582	7632
	Men (%) (2015)	49.2%	49.2%	49.0%
Aging	The number of elderly (aged 65 years or older) (2015)	4092	7311	5671
	The rate of aging (%) (2015)	35.1%	37.5%	36.4%
Disaster damage	The number of people who were dead/missing (mortality, %)	1240 (8.2%)	1773 (7.6%)	753 (4.0%)
	The fatality rate in men (%)	51.0%	45.1%	45.0%
	The mortality of people aged 65 years or older (%)	59.2%	52.1%	63.7%
	The mortality of people aged 65 years or older among 65 years or older population in the 2010 census (%)	9.1%	9.2%	6.2%
	The number of housing damages (the percentage of housing damage, %)	3167 (58.1%)	3341 (44.0%)	3717 (55.4%)
	The number of people living in temporary housing	2146	2168	1990
	The number of people living in a rental apartment except emergency provisional housings by making use of privately-rented housings	129	125	308
Administrative function	Death of Mayer (yes/no)	Yes	No	No
	Damage of city office	Completely damaged	Completely damaged	Partially damaged
	The number of dead or missing who worked in damaged city offices (%)	40 (28%)	111 (25%)	2 (1%)
	The number of care managers per 100,000 who received for long-term care insurance in the people aged 65 years or older (2015)	7787.4	6891.7	8817.6
**Hospital damage**				
Hospitals	The number of hospitals before the disaster	1	2	1
	The number of hospitals that is available to admission after the disaster (2012)	0 (0)	2 (194)	0 (0)
	The number of hospitals that is available to admission after the disaster (2016)	1 (50)	2 (194)	1 (50)
Doctor in Hospitals	The number of doctors before the disaster (2011)	3	6	2
	The number of doctors after the disaster (2016)	5	7	4
	The number of doctors before the disaster in secondary medical areas (2011)	22	45	25
	The number of doctors after the disaster in secondary medical areas (2016)	24	47	34
Clinics	The number of clinics before the disaster	7	9	4
	The number of clinics after the disaster (2012)	4	5	2
	The number of clinics after the disaster in secondary medical areas (2012)	25	38	48
	The number of clinics after the disaster in secondary medical areas (2015)	29	40	49
	The number of clinics that was not available to admission but can treat patients temporary after the disaster in secondary medical areas (bed) (2012)	1 (16)	5 (80)	9 (106)
	The number of clinics that that was not available to admission but can treat patients temporary after the disaster in secondary medical areas (bed) (2015)	2 (21)	5 (80)	7 (85)
	The number of clinics per 100,000 population after the disaster in secondary medical areas (2012)	67.9	97.6	84.0
	The number of clinics per 100,000 population after the disaster in secondary medical areas (2015)	78.8	105.1	78.8
	Damage of referral hospital	Damage	No damage	No damage
Facilities for elderly	The number of long term care facilities (the number of beds) (2015)	3 (206)	3 (280)	2 (180)
	The number of long term care facilities in medical area (the number of beds) (2015)	8 (604)	9 (681)	12 (991)
Functional disability	The number of people receiving services per 100,000 people functional disability in any of the levels above long-term care insurance in secondary medical areas (2015)	24,796.4	23,630.6	21,717.2
	The number of people receiving services per 100,000 people with mild functional disability in care need level 1 or less long-term care insurance in secondary medical areas (2015)	9129.8	7922.0	6002.8
Volunteer	The number of volunteers	78,412	136,706	41,920
Economic status	Income per person (2010)	1797	1873	1768
	Income per person (2011)	1278	1652	1380
	Income per person (2015)	2541	2574	2510
Educational attainment for elderly	Completion of secondary education among people aged 65 years in 2011	45.7%	46.7%	34.7%

Interestingly, the mortality rate of people aged 64 years or younger was highest in Rikuzentakata (47.9%) among the three municipalities. It has been reported that experiencing the loss of a family member is linked to elevated rates of mental health problems that develop later^14^. Indeed, the prevalence of comorbid illness was highest in Rikuzentakata among the municipalities in the 2011 survey (Table 1). However, in contrast to Otsuchi, residents of Rikuzentakata improved over time. Public hospitals and health departments in municipalities had been playing a significant role in providing health services in Iwate Prefecture. While public health facilities were recovering, there were regional differences in the availability of medical and welfare services. Disparities of health are subsequently appearing due to the different approaches for intervention and prevention for health provided by local governments in each medical area^15^. For example, Omama et al. reported that the standard incidence ratio for cerebrovascular diseases differed among medical areas from 2008 to 2016 in Iwate^16^. The municipalities had differences in the coordination functions of municipal administration and in the availability of medical aid and support by volunteers. The improvement of poor SRH in Rikuzentakata might reflect the fact that the town received the highest influx of volunteers from outside out of all of the affected areas in Iwate Prefecture (number of volunteers in Otsuchi: 78,412, compared to Rikuzentakata: 136,706) (Table 3)^17^.

We believe that our detailed case study can provide some lessons for future disasters in other parts of the world. One lesson is that the health of disaster survivors may be linked to not just the physical damage caused by the disaster but also medical systems and other factors. In the present study, we found that it is important for cities, towns, and villages to incorporate medical coordination and cooperation in preparing for disasters. In particular, strengthening cooperation in medical areas beyond the municipal boundaries should be a component of disaster preparedness.

There are several limitations in our study. First, even though we revealed a significant disparity in SRH among municipalities with adjustment for a comprehensive range of subjective (self-reported) and objective indicators, there may have remained some residual differences that we could not take account of, such as educational attainment, household income, and cognitive dysfunction. Ecological data provide some clues in answer to these limitations. For example, according to annual reports provided by the prefectural government, income per person decreased markedly in Otsuchi after the disaster compared to that in other areas (Table 3). With regard to educational attainment, the percentage of people who completed secondary education among people aged 65 years or older in 2011 was not lower in Otsuchi. Second, the participants in this study might have been healthier and had a higher level of health consciousness than people who did not participate in the study. The lower response rate in Otsuchi indicates that our results might have been underestimated (response rates: 15.9% in Otsuchi, 24.7% in Rikuzentakata, and 20.2% in Yamada). Third, some people did not actually live in the three municipalities because they evacuated to other cities such as cities in inland areas in Iwate. However, the impact of mobility is likely to have been small because most people subsequently returned to their original addresses. Fourth, we could not collect access time to health care facilities across the disaster to assess improvement of health care delivery. If we had calculated access time to health care facilities by using, for example, the road network distance between each residential address and the nearest health care facility (using Geographic Information Systems), we could have made more precise estimates of access to facilities. Unfortunately, address information is not available yet in our dataset. Fifth, the number of participants decreased with time during the survey period. Of the participants, 2,140 participants aged 64 years or younger were lost to follow-up by 2015 (participation rate, 59.8%) and 1,578 participants aged 65 years or older were lost to follow-up by 2015 (participation rate, 66.6%). The proportions of participants aged 64 years or younger and aged 65 years or older who completed all 5 waves were 45.7% and 53.8%, respectively, while the proportions who participated in only 1 wave were 20.1% and 11.9%, respectively. Finally, the distribution of prevalences of SRH was not similar to that in a national representative survey. We compared the prevalence of poor SRH in our survey in 2013 and the prevalence of poor SRH in the Comprehensive Survey of Living Conditions in Japan in 2013, in which poor SRH was defined as both “rather poor” and ”poor” out of 5 answers (Supplementary Table 5)^18^. The prevalence of poor SRH in people aged 65 years or older was higher in the Comprehensive Survey of Living Conditions than in our study, while the prevalence of poor SRH in people aged 64 years or younger in that survey was almost the same as that in our study in both sexes. Our results might not be generalizable to a broader sample of elderly Japanese. Although our study is not generalizable to the broader Tohoku region that was directly affected by the earthquake and tsunami, it does offer some advantages compared to other cohort studies, viz., earlier contact with survivors, more frequent follow-up surveys, broader age range, and ability to compare outcomes across three communities within the same prefecture.

In conclusion, we showed differences in the prevalence of poor SRH among municipalities during a 5-year period after a tsunami disaster. The disparities could not be explained by the compositional characteristics of residents in each locality. Our results point to the potential importance of health system characteristics in explaining post-disaster recovery and resilience.

## Materials and methods

The design of the Research Project for Prospective Investigation of Health Problems among Survivors of the Great East Japan Earthquake and Tsunami Disaster (abbreviated to RIAS) has been previously described in detail^19^.

### Ethical consideration

The research plan was approved by the Ethics Committee of Iwate Medical University (approval no. H23-69). The rights and welfare of the participants in this study were protected, and the methods were carried out in accordance with the ethical guidelines outlined in the Declaration of Helsinki. Full details of the study were given to the participants, and they agreed and provided written informed consent for participation in this study.

### Study population

**Figure 3:**
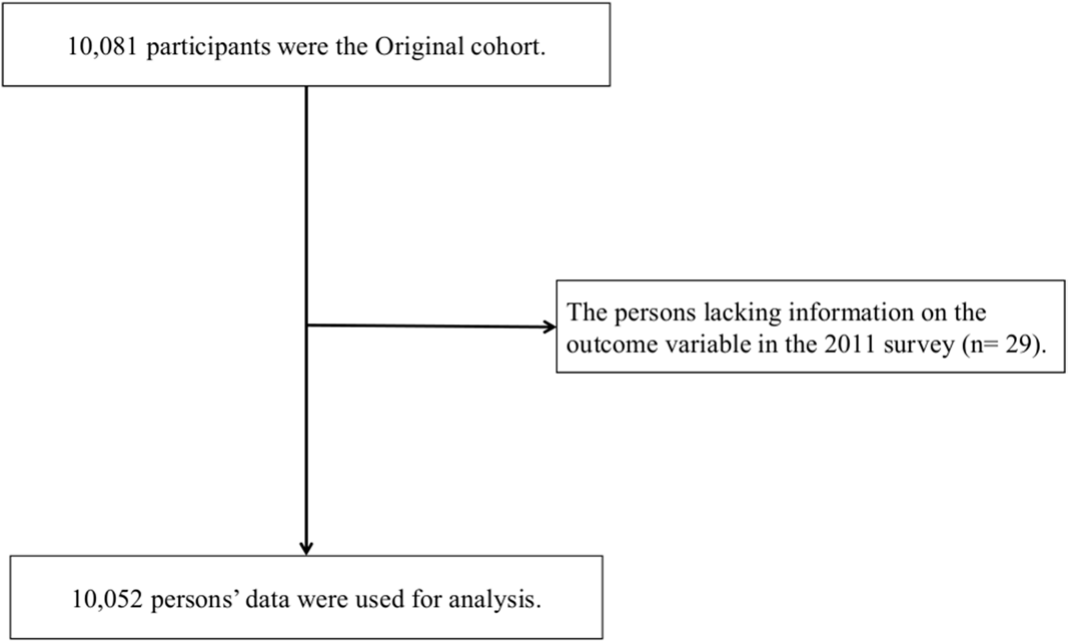
Flow chart of the procedure for selecting patients. Out of 10,081 people in the original cohort, we excluded 29 persons who lacked information on outcome variables in the 2011 survey. Data for 10,052 participants were used for analysis.

In brief, RIAS is an ongoing prospective follow-up study of adults who survived the Great East Japan Earthquake to examine the health impact of the disaster. The survey included the same items as those in annual health check-ups carried out by the National Healthcare Insurance system in Japan, and questions included questions on disaster-related experiences as well as specific questions on functional status in people aged 65 years or older^9^. Individuals aged 18 years or older who were living in the three coastal municipalities of Otsuchi, Rikuzentakata, and Yamada in the southern part of Iwate Prefecture were enrolled in this study (Fig. 2)^5^. Survivors’ addresses were identified from the mandatory residential registration system in each of the municipalities. We recruited all residents aged 18 years or older by announcing about the RIAS survey on community bulletin boards and sending notifications to residents. Informed consent to participate in the survey was obtained from 10,081 residents out of a target sample of 42,831 (participation rate of 23.5%). Participants who had missing information on outcome variables in the 2011 survey were excluded (n = 29). Data for 10,052 participants (mean age, 61.0 years; 39.0% men) were used for analysis (Fig. 3).

### Municipalities

The RIAS study was carried out in Otsuchi Town, Rikuzentakata City, and Yamada Town, which were heavily damaged by the tsunami. The tsunami destroyed central parts of these municipalities including municipal offices and commercial and public facilities including hospitals. The number of people who died or were missing (mortality rate) totaled 1,279 (8.4%) in Otsuchi, 1,808 (7.8%) in Rikuzentakata, and 834 (4.5%) in Yamada^20^. The number of damaged housing amounted to 3,167 (58.1%) in Otsuchi, 3,341 (44.0%) in Rikuzentakata, and 3,717 (55.4%) in Yamada, while the distribution of severity of tsunami damage was similar across municipalities^21^. Mortality rates per 100,000 population before the disaster tended to be similar in the three municipalities (all above the average for Iwate Prefecture as a whole: Otsuchi, 1643.1; Rikuzentakata, 1390.6; Yamada, 1514.7; versus 1184.5 for Iwate prefecture as a whole)^22^.The numbers of residents aged 18 years or older before the disaster were 11,411 in Otsuchi, 18,648 in Rikuzentakata, and 12,772 in Yamada.

### Variables

#### Self-rated health

Self-rated health (SRH) reflects underlying health conditions such as physical dysfunctions and mental abnormalities. People receiving health check-ups have been asked about SRH to evaluate not only an individual’s conditions but also population health outcomes. Since the answer of SRH predicts future mortality and morbidity^23^, it is widely used in screening a high-risk patient. On the other hand, regional differences in poor SRH have been assessed in some countries mainly for determining inequalities of socioeconomic status^24,25^. SRH was assessed by the single question “How do you feel about your health condition?” The four options included “very good,” “good,” “fair,”, and “poor.” We dichotomized the responses into poor SRH (fair and poor) versus the other remaining options (very good and good).

### Covariates

We assessed functional disability, living conditions, self-assessed economic situation (severely distressed vs. not), employment status after the disaster (unemployment vs. not), health habits (smoking status and alcohol drinking status), psychosocial factors (psychological distress, post-traumatic stress disorder (PTSD), and insomnia), social factors (social network and social capital), and disease factors using self-reported questionnaires. Body weight (kg) and height (m) were measured using a stadiometer with a digital scale with light clothes. Waist circumference (cm) was measured. Body mass index (BMI; kg/m^2^) was calculated as body weight (kg) divided by height (m^2^). Blood pressure (mmHg) was measured two times in a sitting position. Non-fasting blood samples were drawn from the antecubital vein in the seated position, and serum levels of high-density lipoprotein cholesterol (mg/dL) and triglycerides (mg/dl), plasma glucose levels (mg/dL) and glycosylated hemoglobin levels (%) were measured.

The Kihon Checklist was used to determine functional disability. The Kihon Checklist consists of simple yes/no questions for 25 items to assess frailty in community-dwelling individuals aged 65 years or older who might require future long-term care insurance in Japan^26^. In accordance with cutoff points validated in a previous study^27^, the participants were categorized into two groups (yes or no) for low levels of instrumental activities of daily living (IADL): low level of physical strength, malnutrition, low level of oral function, home-bound, and cognitive impairment. In addition, we defined persons who fulfilled ≥ 10 risk factors in 20 out of 25 items as persons with general frailty^28^. The living conditions of the participants were dichotomized to living in prefabricated temporary housing or other types of accommodation. Smoking status was binarized (current smokers vs. non-current smokers), and alcohol drinking status was similarly classified into two categories (drinkers vs. non-drinkers). With regard to psychological factors, psychological distress was assessed by the Japanese version of the K6 scale^29,30^. Based on a previous study, the participants were classified as having severe psychological distress (scores of 13 +), moderate psychological distress (5–12), or no psychological distress (0–4)^19^. Symptoms of PTSD were assessed by the question “Have you had the following experiences related to the disaster more than twice in the past week?” with 3 possible response items: (1) I had a bad dream or remember the disaster experience even though I do not remember it; (2) I get terribly upset when I remember the disaster experience; (3) When I recall experiences, I get a physical reaction (chest tightness, dyspnea, sweat, vertigo, etc.). Based on a previous validation study, participants were classified into those exhibiting PTSD symptoms (more than 1 item) versus those with no PTSD symptoms (zero items)^31^. Insomnia was defined as scores of 6 to 24 on the Athens Insomnia Scale (AIS)^32–35^. Social network was evaluated by Lubben’s Social Network Scale^36,37^. Social capital was assessed using 4 questions on social cohesion including residents’ perceptions of trust in the community and levels of mutual help^38^. For determination of disease factors, participants were asked about present illnesses and symptoms. Present illnesses were assessed from 21 options: stroke, hypertension, myocardial infarction or angina pectoris, kidney diseases, liver diseases, diabetes mellitus, peptic ulcer disease, tuberculosis and/or pleurisy, arthralgia, osteoporosis, cancer, dyslipidemia, asthma and/or emphysema and/or chronic bronchitis, anemia, dental diseases, diseases specified by the Japanese government as being worrisome, having no known treatment and having unknown causes, allergy, physical disability certification, receiving dialysis, women during pregnancy, and others. With regard to symptoms, participants were asked “Have you recently had any symptoms due to diseases or injuries?”. We classified the participants into two categories: those with symptoms and those with no symptoms.

We also assessed cardiovascular risk factors using biological measurements. Overweight was defined as BMI of ≥ 25 kg/m^2^. Diabetes mellitus was defined as plasma glucose level ≥ 200 mg/dL, plasma glycosylated hemoglobin level (NSGP) ≥ 6.5%, or a diagnosis of diabetes mellitus reported on the questionnaire. Metabolic syndrome was defined according to the criteria of the International Diabetes Federation^39^.

### Statistical analyses

All analyses were stratified by age group, 64 years or younger and 65 years or older, considering the differences in prevalence of poor SRH and covariates for analyses. Although no significant interactions were found between age groups (*p* for interaction, 0,293 (≥ 0.20)), substantial differences in characteristics were seen in relation to poor SRH by age group. Baseline characteristics were compared among the three municipalities in the 2011 survey. Categorical variables are expressed as percentages, and continuous variables were expressed as means ± standard deviation (SD). Differences in proportions/means among the three municipalities were tested using the chi-squared test (categorical variables) or analysis of variance with Bonferroni correction (continuous variables).

We analyzed poor SRH across four follow-up examinations using generalized linear mixed effect models. Repeated measures within individuals were modeled adopting a first-order autoregressive (AR-1) covariance structure. A sequence of three models was run. In Model 1, we included explanatory variables including age, sex, survey year, municipalities, and an interaction between municipalities × survey year as fixed effects. Among individuals aged 65 years or older in Model 2, we additionally adjusted for functional disability. In Model 3, we further adjusted for living conditions, self-assessed economic situation, employment status, health habits, psychosocial factors, social factors, and diseases factors in both age groups. We defined all independent variables as categorical variables except for age. We showed two patterns in the results: the odds ratios (ORs) for poor SRH and the prevalence of poor SRH during the survey period among municipalities estimated from these models. As sensitivity analysis, the same statistical analyses were conducted with adjustment for some covariates as discrete variables including present illness (0 to 21), symptoms (0 to 21), and general frailty scores (0 to 20) (Model 4). We also performed the same analysis with the missing covariate data in the 2011 study by inverse probability weighting.

Statistical analyses were performed using SPSS version 25•0 (IBM Corp., Armonk, NY, USA). All statistical tests were two-sided, and analysis items with *p*-values < 0.05 were considered statistically significant.

## Supplementary Information

Supplementary Information.

## Data Availability

Data are available from the authors upon reasonable request and with permission of Research Project for Prospective Investigation of Health Problems Among Survivors of the Great East Japan Earthquake and Tsunami Disaster (RIAS) (Iwate Medical University).
