# Supplementary material for: The differences of poor SRH among municipalities in Iwate after the Great East Japan Earthquake
Source: Sci Rep. 2021 Aug 26;11:17270. doi: 10.1038/s41598-021-96237-3 (PMC8390508; doi:10.1038/s41598-021-96237-3)
Supplement: Supplementary file 1 — Supplementary Information. [file 41598_2021_96237_MOESM1_ESM.pdf]

Supplementary Materials

The differences of poor SRH among municipalities in Iwate after the Great East Japan Earthquake

Shuko Takahashi; Haruki Shimoda; Kiyomi Sakata; Akira Ogawa; Seiichiro Kobayashi; Ichiro Kawachi

Correspondence to: shutakahashi-iwt@umin.ac.jp

Supplementary Table S1.
Comparison of characteristics of the analytic sample at baseline and the whole population in the 2011 local census in municipalities.

Supplementary Table S2.
Comparison of poor self-rated health among the municipalities with the discrete of present illness, symptoms, and general frailty score using generalized mixed effect models

Supplementary Table S3.
Comparison of poor self-rated health among the municipalities using inverse propensity weighting in generalized mixed effect models

Supplementary Table S4.
Characteristics of people aged 65 years or older in the 2011 and 2015 survey

Supplementary Table S5.
Comparison of the prevalence of poor self-rated health in the Comprehensive survey of Living Conditions in Japan in 2013 and the prevalence of poor self-rated health in the present study

Supplementary Table S1. Comparison of characteristics of the analytic sample at baseline and the whole population in the 2011 local census in municipalities.

		2011 local census	2011 all analytic sample
		n (%)	n (%)
Sex	Men	19180 (46.2)	3925 (39.0)
	Women	22354 (53.8)	6127 (61.0)
Age classes	20-29 year	2697 (6.5)	304 (3.0)
	30-39 year	4916 (11.8)	737 (7.4)
	40-49 year	6011 (14.5)	1119 (11.2)
	50-59 year	6956 (16.7)	1541 (15.4)
	60-69 year	8500 (20.5)	3054 (30.5)
	70-79 year	7506 (18.1)	2611 (26.1)
	80 year or older	4948 (11.9)	655 (6.5)

Supplementary Table S2. Comparison of poor self-rated health among the municipalities with the discrete variables of present illness, symptoms, and general frailty score using generalized mixed effect models

	64 years or younger		65 years or older	
	Model 4 (n=5052)		Model 4 (n=4481)	
	Odds ratio	95% CI	Odds ratio	95% CI
Intercept	0.03	0.02, 0.05	0.06	0.02, 0.15
Age	1.00	0.99, 1.00	0.99	0.98, 1.00
Sex (men)	1.19	1.00, 1.41	1.55	1.32, 1.82
Otsuhi (ref; Yamada)	0.95	0.73, 1.24	0.91	0.67, 1.23
Rikuzentakata	0.86	0.69, 1.06	0.99	0.78, 1.26
2011	Base		Base	
2012	0.93	0.73, 1.18	0.81	0.60, 1.09
2013	0.81	0.63, 1.03	1.06	0.80, 1.41
2014	1.00	0.77, 1.28	1.02	0.76, 1.36
2015	0.78	0.60, 1.02	1.02	0.75, 1.38
2012 × Otsuchi (ref; 2011 × Yamada)	1.00	0.67, 1.48	1.29	0.83, 2.00
2012 × Rikuzentakata	1.19	0.86, 1.63	1.39	0.98, 1.96
2013 × Otsuchi	1.53	1.03, 2.28	1.55	1.02, 2.35
2013 × Rikuzentakata	1.20	0.87, 1.67	1.06	0.76, 1.48
2014 × Otsuchi	1.52	1.04, 2.22	1.57	1.03, 2.40
2014 × Rikuzentakata	0.99	0.71, 1.38	1.32	0.94, 1.84
2015 × Otsuchi	1.28	0.85, 1.92	1.98	1.30, 3.02
2015 × Rikuzentakata	1.12	0.80, 1.58	1.18	0.84, 1.66
General frailty			1.14	1.10, 1.18
Low levels of IADL			0.97	0.82, 1.14
Low level of physical strength			1.45	1.23, 1.71
Malnutrition			1.16	1.01, 1.33
Low level of oral function			0.86	0.74, 1.01
Home-bound			0.84	0.68, 1.03
Cognitive impairment			0.83	0.73, 0.95
Living in prefabricated temporary housing	0.99	0.87, 1.14	1.01	0.88, 1.16
Severely distressed economic situation	1.18	1.04, 1.34	1.08	0.96, 1.22
Unemployment (2011)	0.84	0.72, 0.98	0.75	0.61, 0.92
Current smokers	1.21	1.02, 1.43	1.00	0.79, 1.28
Drinkers	0.80	0.70, 0.93	0.80	0.68, 0.94
Moderate psychological distress	1.56	1.37, 1.77	1.45	1.28, 1.64
Severe psychological distress	2.55	2.02, 3.21	1.78	1.40, 2.27
PTSD symptom	0.98	0.86, 1.12	1.01	0.90, 1.13
Insomnia	2.36	2.08, 2.67	1.99	1.75, 2.26

**Low level of social network**	1.13	1.01, 1.27	1.14	1.02, 1.28
**Low level of social capital**	1.11	0.94, 1.32	1.08	0.92, 1.27
**Having present illness**	1.58	1.46, 1.71	1.22	1.15, 1.30
**Having symptoms**	1.39	1.35, 1.43	1.38	1.34, 1.42
**Obesity**	0.97	0.83, 1.12	0.94	0.83, 1.08
**Diabetes mellitus**	1.02	0.81, 1.28	1.01	0.85, 1.20
**Metabolic syndrome**	1.09	0.94, 1.28	0.98	0.85, 1.12

Abbreviations: CI, confidence interval; IADL, instrumental activities of daily living, PTSD, post-traumatic stress disorder

The number of participants represents the individuals who participated in at least one survey from 2011 to 2015 and had all variables adjusted in each model in one's participated survey.

Supplementary Table S3. Comparison of poor self-rated health among the municipalities using inverse propensity weighting in generalized mixed effect models

	64 years or younger			
	Model 1 (n=5327)		Model 2 (n=5052)	
	Odds ratio	95% CI	Odds ratio	95% CI
Intercept	0.12	0.07, 0.19	0.01	0.01, 0.02
Age	1.01	1.00, 1.02	1.00	0.99, 1.01
Sex (men)	0.90	0.72, 1.12	1.26	1.00, 1.60
Otsuhi (ref; Yamada)	1.08	0.77, 1.52	0.99	0.76, 1.28
Rikuzentakata	0.79	0.60, 1.06	0.81	0.65, 1.00
2011	Base		Base	
2012	0.82	0.63, 1.06	1.01	0.72, 1.42
2013	0.63	0.46, 0.87	0.77	0.52, 1.15
2014	0.75	0.55, 1.02	1.33	0.90, 1.96
2015	0.59	0.41, 0.86	0.97	0.65, 1.44
2012 × Otsuchi (ref; 2011 × Yamada)	1.08	0.69, 1.68	0.80	0.45, 1.42
2012 × Rikuzentakata	1.05	0.74, 1.49	1.04	0.67, 1.60
2013 × Otsuchi	1.32	0.83, 2.08	1.33	0.73, 2.43
2013 × Rikuzentakata	1.42	0.94, 2.15	1.60	0.98, 2.61
2014 × Otsuchi	1.37	0.86, 2.19	1.13	0.65, 1.98
2014 × Rikuzentakata	1.21	0.79, 1.84	1.18	0.72, 1.94
2015 × Otsuchi	1.28	0.77, 2.12	0.87	0.47, 1.59
2015 × Rikuzentakata	1.17	0.73, 1.87	1.13	0.70, 1.82
General frailty				
Low levels of IADL				
Low level of physical strength				
Malnutrition				
Low level of oral function				
Home-bound				
Cognitive impairment				
Living in prefabricated temporary housing			1.06	0.86, 1.31
Severely distressed economic situation			1.25	1.05, 1.50
Unemployment (2011)			0.82	0.66, 1.03
Current smokers			1.26	1.00, 1.60
Drinkers			0.80	0.64, 0.98
Moderate psychological distress			1.68	1.39, 2.02
Severe psychological distress			3.55	2.64, 4.78
PTSD symptom			1.30	1.05, 1.61
Insomnia			2.13	1.76, 2.57

Low level of social network	1.21	1.01, 1.44
Low level of social capital	1.19	0.98, 1.44
Having present illness	2.19	1.79, 2.67
Having symptoms	4.48	3.59, 5.58
Obesity	0.84	0.68, 1.04
Diabetes mellitus	1.13	0.85, 1.51
Metabolic syndrome	1.15	0.92, 1.44

21

	65 years or older					
	Model 1 (n=4725)		Model 2 (n=4662)		Model 3 (n=4481)	
	Odds ratio	95% CI	Odds ratio	95% CI	Odds ratio	95% CI
Intercept	0.02	0.01, 0.05	0.12	0.04, 0.34	0.02	0.01, 0.05
Age	1.04	1.02, 1.05	1.00	0.99, 1.02	1.00	0.98, 1.01
Sex (men)	0.93	0.79, 1.10	1.07	0.91, 1.26	1.56	1.28, 1.90
Otsuhi (ref; Yamada)	1.02	0.76, 1.37	1.01	0.74, 1.40	0.99	0.73, 1.34
Rikuzentakata	0.95	0.74, 1.22	0.96	0.74, 1.24	1.02	0.80, 1.30
2011	Base		Base		Base	
2012	0.83	0.66, 1.04	0.78	0.59, 1.02	0.82	0.59, 1.14
2013	0.90	0.69, 1.18	0.92	0.69, 1.23	1.08	0.76, 1.54
2014	0.91	0.70, 1.17	0.90	0.67, 1.23	1.03	0.73, 1.46
2015	0.82	0.62, 1.08	0.85	0.61, 1.17	1.01	0.70, 1.46
2012 × Otsuchi (ref; 2011 × Yamada)	0.85	0.59, 1.22	0.98	0.65, 1.48	1.21	0.73, 1.99
2012 × Rikuzentakata	1.11	0.84, 1.48	1.32	0.94, 1.84	1.39	0.94, 2.05
2013 × Otsuchi	1.14	0.78, 1.67	1.37	0.90, 2.09	1.45	0.87, 2.40
2013 × Rikuzentakata	0.97	0.70, 1.33	1.20	0.84, 1.70	1.04	0.69, 1.56
2014 × Otsuchi	1.23	0.85, 1.79	1.43	0.93, 2.20	1.35	0.80, 2.30
2014 × Rikuzentakata	0.99	0.73, 1.35	1.34	0.94, 1.91	1.36	0.91, 2.02
2015 × Otsuchi	1.57	1.07, 2.30	1.70	1.10, 2.62	1.50	0.89, 2.53
2015 × Rikuzentakata	1.08	0.78, 1.49	1.28	0.88, 1.86	1.14	0.75, 1.74
General frailty			1.27	0.97, 1.67	1.20	0.84, 1.70
Low levels of IADL			1.19	1.02, 1.39	1.26	1.05, 1.52
Low level of physical strength			2.08	1.79, 2.43	2.00	1.68, 2.37
Malnutrition			1.51	1.32, 1.72	1.41	1.19, 1.66
Low level of oral function			1.62	1.40, 1.87	1.19	1.01, 1.41
Home-bound			1.18	0.96, 1.46	1.07	0.85, 1.36
Cognitive impairment			1.21	1.08, 1.36	0.97	0.85, 1.11
Living in prefabricated temporary housing					0.98	0.82, 1.16
Severely distressed economic situation					1.22	1.05, 1.42
Unemployment (2011)					0.74	0.57, 0.94
Current smokers					1.09	0.82, 1.44
Drinkers					0.86	0.69, 1.08

Moderate psychological distress	1.51	1.29, 1.76
Severe psychological distress	2.38	1.76, 3.21
PTSD symptom	1.08	0.92, 1.26
Insomnia	2.18	1.87, 2.52
Low level of social network	1.20	1.05, 1.38
Low level of social capital	1.09	0.89, 1.32
Having present illness	1.90	1.51, 2.40
Having symptoms	4.38	3.75, 5.13
Obesity	1.00	0.85, 1.18
Diabetes mellitus	1.30	1.05, 1.61
Metabolic syndrome	0.98	0.83, 1.15

Abbreviations: CI, confidence interval; IADL, instrumental activities of daily living, PTSD, post-traumatic stress disorder

The number of participants represents the individuals who participated in at least one survey from 2011 to 2015 and had all variables adjusted in each model in one's participated survey.

Supplementary Table S4. Characteristics of people aged 65 years or older in the 2011 and 2015 survey

		65 years or older (n=4725)				
		2011				
		Missing	Otsuchi (n=1054)	Rikuzentakata (n=2351)	Yamada (n=1922)	*P* value
		n (%)	n (%)	n (%)	n (%)	
Functional disability	General frailty	332 (7.0)	63 (6.9)	115 (4.9)	52 (4.6)	0.034*
	Lower IADL	57 (1.2)	268 (27.3)	454 (18.3)	292 (24.2)	<0.001*
	Low physical strength	113 (2.4)	171 (17.7)	485 (19.8)	196 (16.4)	0.042*
	Malnutrition	46 (1.0)	299 (30.5)	599 (24.0)	376 (31.3)	<0.001*
	Low oral function	60 (1.3)	137 (14.0)	405 (16.3)	172 (14.3)	0.143
	Home-bound	8 (0.2)	110 (11.1)	275 (11.0)	141 (11.6)	0.828
	Cognitive impairment	58 (1.2)	424 (43.1)	1078 (43.4)	509 (42.4)	0.838
Living conditions	Living in temporary housing	81 (1.7)	350 (35.9)	752 (30.4)	354 (29.5)	0.003*
Socioeconomic status	Severely distressed economic situation	26 (0.6)	430 (43.5)	1092 (43.6)	506 (42.0)	0.655
Health habits	Current smokers	0 (0.0)	100 (10.1)	214 (8.5)	97 (8.0)	0.201
	Drinkers	0 (0.0)	296 (29.7)	667 (26.5)	375 (30.8)	0.013*
Psychological factors	Moderate psychological distress	98 (2.1)	326 (33.6)	799 (32.4)	392 (32.9)	0.197
	Severe psychological distress		52 (5.4)	123 (5.0)	81 (6.8)	
	PTSD Symptom	44 (0.9)	462 (47.0)	1239 (49.8)	531 (43.9)	0.003*
	Insomnia	79 (1.7)	289 (29.8)	739 (29.8)	410 (34.3)	0.016*
Social factors	Low level of social network	121 (2.6)	383 (39.7)	886 (36.1)	460 (38.7)	0.097
	Low level of social capital	20 (0.4)	93 (9.4)	191 (7.6)	123 (10.1)	0.024*
Disease factors	Having present illness	0 (0.0)	804 (80.8)	2043 (81.3)	915 (75.2)	<0.001*
	Having symptoms	85 (1.8)	365 (37.7)	1120 (45.3)	505 (42.2)	<0.001*
Cardiovascular risk factors	Obesity	332 (7.0)	397 (39.9)	796 (31.7)	416 (34.2)	<0.001*
	Diabetes mellitus	113 (2.4)	134 (13.5)	341 (13.6)	153 (12.6)	0.690
	Metabolic syndrome	46 (1.0)	312 (31.4)	785 (31.2)	360 (29.6)	0.545

		65 years or older (n=4725)				
		2015				
		Missing	Otsuchi (n=1054)	Rikuzentakata (n=2351)	Yamada (n=1922)	*P* value
		n (%)	n (%)	n (%)	n (%)	
Functional disability	General frailty	1564 (33.1)	26 (4.3)	44 (2.7)	29 (3.7)	0.142
	Lower IADL	1712 (36.2)	149 (22.7)	260 (15.5)	155 (18.4)	<0.001*
	Low physical strength	1598 (33.8)	133 (20.5)	312 (18.6)	162 (19.4)	0.578
	Malnutrition	1544 (32.7)	110 (17.0)	190 (11.5)	180 (21.6)	<0.001*
	Low oral function	1523 (32.2)	99 (15.1)	301 (17.9)	149 (17.6)	0.252
	Home-bound	1557 (33.0)	41 (6.2)	12 (0.7)	52 (6.1)	<0.001*
	Cognitive impairment	1544 (32.7)	239 (36.4)	459 (27.5)	305 (36.3)	<0.001*
Living conditions	Living in temporary housing	1529 (32.4)	149 (22.4)	259 (15.4)	177 (20.8)	<0.001*
Socioeconomic status	Severely distressed economic situation	1523 (32.2)	634 (95.6)	1639 (97.3)	830 (97.2)	0.1
Health habits	Current smokers	1578 (33.4)	48 (7.3)	105 (6.4)	47 (5.6)	0.398
	Drinkers	1578 (33.4)	176 (26.9)	428 (25.9)	233 (27.8)	0.595
Psychological factors	Moderate psychological distress	1560 (33.0)	153 (23.6)	367 (21.9)	162 (19.3)	0.364
	Severe psychological distress		15 (2.3)	43 (2.6)	22 (2.6)	
	PTSD Symptom	1532 (32.4)	165 (25.0)	357 (21.2)	244 (28.7)	<0.001*
	Insomnia	1566 (33.1)	148 (22.6)	377 (22.5)	136 (16.4)	0.001*
Social factors	Low level of social network	1580 (33.4)	138 (21.5)	291 (17.3)	174 (21.1)	0.020*
	Low level of social capital	1553 (32.9)	80 (12.3)	145 (8.6)	117 (14.0)	<0.001*
Disease factors	Having present illness	1512 (32.0)	584 (87.6)	1492 (88.3)	713 (83.2)	0.001*
	Having symptoms	1569 (33.2)	298 (45.7)	831 (49.3)	355 (43.3)	0.015 *
Cardiovascular risk factors	Obesity	1564 (33.1)	240 (36.6)	528 (31.9)	280 (33.4)	0.097
	Diabetes mellitus	1598 (33.8)	94 (14.4)	279 (16.9)	119 (14.2)	0.131
	Metabolic syndrome	1544 (32.7)	227 (34.7)	602 (36.4)	286 (34.1)	0.469

Categorical variables are presented as the number of cases (%).
P values were calculated using the chi-squared test for categorical variables.
* Statistically significant differences among municipalities.
Abbreviations: IADL, instrumental activities of daily living, PTSD, post-traumatic stress disorder

Supplementary Table S5. Comparison of the prevalence of poor self-rated health in the Comprehensive survey of Living Conditions in Japan in 2013 and the prevalence of poor self-rated health in the present study

	Men		Women	
	Comprehensive Survey of Living Conditions	Present study	Comprehensive Survey of Living Conditions	Present study
	%	%	%	%
20-29 year	6.6	6.5	8.1	14.0
30-39 year	8.4	14.2	10.4	11.5
40-49 year	10.3	9.8	12.6	14.9
50-59 year	12.7	12.2	14.4	12.2
60-69 year	15.0	12.7	14.9	12.9
70-79 year	22.4	14.9	23.8	18.7
80 year or older	35.6	23.8	36.4	17.6
65 years or older	27.2	15.7	25.3	17.0
